# Simultaneous Determination of Dexamethasone, Ondansetron, Granisetron, Tropisetron, and Azasetron in Infusion Samples by HPLC with DAD Detection

**DOI:** 10.1155/2017/6749087

**Published:** 2017-01-11

**Authors:** Fu-chao Chen, Lin-hai Wang, Jun Guo, Xiao-ya Shi, Bao-xia Fang

**Affiliations:** ^1^Department of Pharmacy, Dongfeng Hospital, Hubei University of Medicine, Shiyan, Hubei 442008, China; ^2^Department of Pharmacy, Renmin Hospital, Hubei University of Medicine, Shiyan, Hubei 442000, China; ^3^Department of Oncology, Dongfeng Hospital, Hubei University of Medicine, Shiyan, Hubei 442008, China

## Abstract

A simple and rapid high-performance liquid chromatography with diode array detector (HPLC-DAD) method has been developed and validated for simultaneous quantification of five antiemetic agents in infusion samples: dexamethasone, ondansetron, granisetron, tropisetron, and azasetron. The chromatographic separation was achieved on a Phenomenex C_18_ column (4.6 mm × 150 mm, 5 *μ*m) using acetonitrile-50 mM KH_2_PO_4_ buffer-triethylamine (25 : 74 : 1; v/v; pH 4.0). Flow rate was 1.0 mL/min with a column temperature of 30°C. Validation of the method was made in terms of specificity, linearity, accuracy, and intra- and interday precision, as well as quantification and detection limits. The developed method can be used in the laboratory to routinely quantify dexamethasone, ondansetron, granisetron, tropisetron, and azasetron simultaneously and to evaluate the physicochemical stability of referred drugs in mixtures for endovenous use.

## 1. Introduction

**Figure 1:**
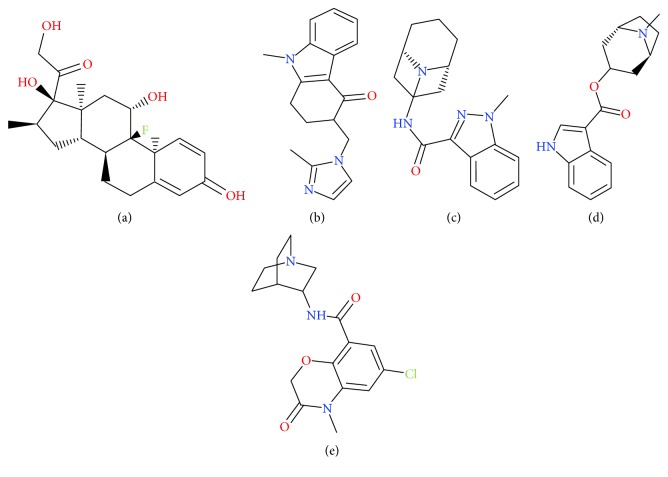
Chemical structure of DEX (a), OND (b), GRA (c), TRO (d), and AZA (e).

Dexamethasone (DEX) (Figure 1(a)), a synthetic corticosteroid, has long been used as antiemetic agent in patients undergoing cancer chemotherapy [1, 2], being effective for both acute and delayed nausea and vomiting. Selective 5-hydroxytryptamine type 3 (5-HT_3_) receptor antagonists, including ondansetron (OND) (Figure 1(b)), granisetron (GRA) (Figure 1(c)), tropisetron (TRO) (Figure 1(d)), and azasetron (AZA) (Figure 1(e)), are effective and potent antiemetic drugs which are recommended by clinical practice guidelines for patients undergoing surgery and cancer chemotherapy [3–6]. Literature survey has revealed that coadministration of antiemetics from different classes could be a more effective antiemetic treatment modality, and DEX is a standard component of antiemetic combination regimens with 5-HT_3_ antagonists [3, 7–10]. Hence, mixtures of 5-HT_3_ receptor antagonists, alone or in combination with DEX, are often used in clinical practice to relieve chemotherapy induced nausea and vomiting. However, there are no commercially available such drug mixtures, and they must be prepared in the hospital pharmacy departments for clinical use.

From the literature survey it is evident that various analytical methods were available for the determination of DEX, OND, GRA, TRO, and AZA with other combinations by using high-performance liquid chromatography (HPLC) [11–31]. No method has been reported in the literature for simultaneous estimation of DEX, OND, GRA, TRO, and AZA in infusion samples. Hence, the objective of the current study was to develop and validate a simple, rapid, accurate, and precise HPLC method for simultaneous estimation of DEX, OND, GRA, TRO, and AZA in infusion samples.

## 2. Experimental

### 2.1. Chemicals and Reagents

The working standards of DEX sodium phosphate, OND hydrochloride, GRA hydrochloride, TRO hydrochloride, and AZA hydrochloride were purchased from the National Institutes for Food and Drug Control (Beijing, China) and stored at 4°C. The pharmaceutical formulations used in this study were DEX sodium phosphate injection 5 mg/1 mL (Cisen Pharmaceutical Co., Ltd., Shandong, China), OND hydrochloride injection 4 mg/2 mL (Qilu Pharmaceutical Co., Ltd., Shandong, China), GRA hydrochloride injection 3 mg/3 mL (Cinkate Pharmaceutical Corporation, Suzhou, China), TRO hydrochloride injection 5 mg/1 mL (Qilu Pharmaceutical Co., Ltd., Shandong, China), and AZA hydrochloride injection 10 mg/2 mL (Wanma Pharmaceutical Co., Ltd., Zhejiang, China). The solution of 0.9% NaCl used to prepare the sample mixtures was from Kelun Pharmaceutical Co., Ltd. (Sichuan, China). Potassium dihydrogen phosphate KH_2_PO_4_, triethylamine, and phosphoric acid of AR Grade were obtained from Xilong Chemical Ltd. (Guangdong, China). HPLC grade acetonitrile was purchased from Fisher Scientific International (St Louis, MO, USA). Ultrapure water was purified using a Milli-Q system (Millipore, Bedford, MA, USA).

### 2.2. HPLC Instrumentation and Chromatographic Conditions

An UltiMate® 3000 standard high pressure liquid chromatographic instrument (Dionex, Germany) composed of an UltiMate 3000 quaternary gradient pump, an ASI-100 autosampler, a TCC-100 thermostat column oven, and an ultraviolet detector (DAD) was employed in the study. Data acquisition was carried out using Chromeleon® software. Chromatographic analyses were performed on a Phenomenex C_18_ column (4.6 mm × 150 mm, 5 *μ*m) and using the mobile phase of acetonitrile-50 mM KH_2_PO_4_ buffer-triethylamine (25 : 74 : 1; pH adjusted to 4.0 using diluted phosphoric acid). The mobile phase was prepared daily and filtered through a 0.22 *μ*m membrane filter (Millipore Corp., USA). The flow rate of the mobile phase was kept at 1.0 mL/min. The selected detection wavelengths for DEX, OND, GRA, TRO, and AZA were 241 nm, 310 nm, 302 nm, 285 nm, and 302 nm, respectively. The assay was performed at 30°C and injection volume was 20 *μ*L.

### 2.3. Preparation of Stock and Working Solutions

We accurately weighed and transferred 20 mg of DEX sodium phosphate, 16 mg of OND hydrochloride, 12 mg of GRA hydrochloride, 10 mg of TRO hydrochloride, and 20 mg of AZA hydrochloride working standard into a 100 mL volumetric flask. We added about 70 mL of deionised water and used sonication for dissolving completely and made volume up to the mark with the same solvent to obtain the final concentration of 0.2 mg/mL of DEX sodium phosphate, 0.16 mg/mL of OND hydrochloride, 0.12 mg/mL of GRA hydrochloride, 0.1 mg/mL of TRO hydrochloride, and 0.2 mg/mL of AZA hydrochloride, respectively. The solutions were kept at –20°C until use. Fresh working standard solutions were prepared by diluting the stock solution with deionised water to the required concentrations before use.

### 2.4. Preparation of Infusion Samples

In order to mimic a concentration range relevant to clinical practice and the conditions commonly occurring in hospitals, four sample infusion solutions were prepared under aseptic conditions in laminar flow hoods.

**Solution 1 .**

2 mL (10 mg) DEX sodium phosphate injectable solution and 4 mL (8 mg) OND hydrochloride injectable solution were transferred to a 100 mL polyolefin bags and filled with 0.9% sodium chloride injection.

**Solution 2 .**

2 mL (10 mg) DEX sodium phosphate injectable solution and 3 mL (3 mg) GRA hydrochloride injectable solution were transferred to a 100 mL polyolefin bags and filled with 0.9% sodium chloride injection.

**Solution 3 .**

2 mL (10 mg) DEX sodium phosphate injectable solution and 1 mL (5 mg) TRO hydrochloride injectable solution were transferred to a 100 mL polyolefin bags and filled with 0.9% sodium chloride injection.

**Solution 4 .**

2 mL (10 mg) AZA hydrochloride injectable solution was transferred to a 100 mL polyolefin bags and filled with 0.9% sodium chloride injection.

### 2.5. Validation of the Method

The developed analytical method was subjected to validation with respect to various parameters such as linearity, intra- and interday precision, accuracy, limit of quantification (LOQ), limit of detection (LOD), and reproducibility for each analyte.

### 2.6. Physicochemical Stability Study of the Infusion Samples

The compatibility and stability studies of the infusion samples were performed at 25 ± 0.5°C; all the solutions were protected from light exposure and checked at predetermined times: 0, 2, 4, 8, 24, and 48 hours. At the specified times, the infusion samples were examined for the changes in appearance and the pH value of the mixture was also determined in a digital Crison phs-3c pH meter. Moreover, the concentrations of the drugs were determined at each analysis by the above described HPLC-DAD method. In the concentrations' analysis, 2 mL samples were collected from each polyolefin bag and were diluted 1 : 5 in deionised water before injection into HPLC system so as to be within the range covered by the calibration curves.

## 3. Results and Discussion

### 3.1. Optimization of Chromatographic Conditions

**Figure 2:**
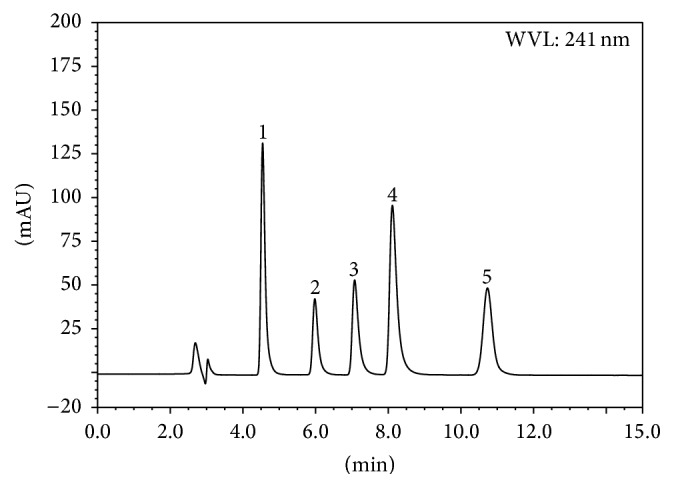
HPLC chromatograms of AZA (1), GRA (2), TRO (3), OND (4), and DEX (5).

The present study is aimed at developing a chromatographic system capable of eluting the individual drugs, permitting their separation and simultaneous determination in infusion solutions. On the basis of the literature consulted [11–31], an acidic aqueous medium and acetonitrile were selected to start the optimization of mobile phase composition. Trials showed that an acidic mobile phase with reverse phase C_18_ column gives symmetric and sharp peaks. The best resolution and analysis time was obtained through isocratic elution using a mobile phase consisting of acetonitrile-50 mM KH_2_PO_4_ buffer-triethylamine (25 : 74 : 1; v/v; pH 4.0) at a flow rate of 1.0 mL/min. Buffer pH was evaluated in the range from 2.0 to 6.0, and good resolution and peak shapes were achieved at pH 4.0.  Figure 2 displays representative HPLC profiles of mixtures detected at 241 nm for the five components. Under these chromatographic conditions, the average retention time for DEX sodium phosphate, OND hydrochloride, GRA hydrochloride, TRO hydrochloride, and AZA hydrochloride was found to be 10.72, 8.12, 5.97, 7.10, and 4.55 min, respectively. DAD was used because it has advantages over conventional UV detection. It enables peak purity to be checked and simultaneous recording at the wavelength of maximum absorbance. The absorption maximum of DEX sodium phosphate, OND hydrochloride, GRA hydrochloride, TRO hydrochloride, and AZA hydrochloride at 241, 310, 302, 285, and 302 nm was selected for detection.

### 3.2. Linearity, Limit of Detection (LOD), and Limit of Quantitation (LOQ)

Linearity was performed by preparing mixed standard solutions of DEX sodium phosphate, OND hydrochloride, GRA hydrochloride, TRO hydrochloride, and AZA hydrochloride at six concentration levels. The linearity of detector response for DEX sodium phosphate, OND hydrochloride, GRA hydrochloride, TRO hydrochloride, and AZA hydrochloride was demonstrated by prepared solutions of over the concentration range of 1.0–100.0 *μ*g/mL, 2.0–80.0 *μ*g/mL, 1.2–36.0 *μ*g/mL, 1.0–50.0 *μ*g/mL, and 2.0–100.0 *μ*g/mL, respectively. The peak area ratio of each sample against respective concentration of five antiemetic agents was found to be linear. The correlation coefficient for all five antiemetic agents was greater than 0.998. Linearity results were presented in Table 1. The LOD and LOQ for five antiemetic agents were determined based on the standards/baseline signal-to-noise (*S*/*N*) ratio. Dilutions and injections of DEX sodium phosphate, OND hydrochloride, GRA hydrochloride, TRO hydrochloride, and AZA hydrochloride standards were then made until an HPLC chromatograph showed that the three-peak height reached an *S*/*N* of approximately 10 : 1 and 3 : 1 for LOQ and LOD solutions, respectively. The LOD and LOQ for both drugs were determined and shown in Table 1.

### 3.3. Precision

The precision (RSD) of the method was determined as intraday precision and intermediate precision. Intraday precision was estimated by assaying quality control samples at three concentration levels (1.0, 20.0, and 40.0 *μ*g/mL for DEX sodium phosphate; 2.0, 16.0, and 32.0 *μ*g/mL for OND hydrochloride; 1.2, 6.0, and 12.0 *μ*g/mL for GRA hydrochloride; 1.0, 10.0, and 20.0 *μ*g/mL for TRO hydrochloride; 2.0, 20.0, and 40.0 *μ*g/mL for AZA hydrochloride) with six determinations per concentration at the same day. Interday precision (6 days) was also estimated as the RSD calculated from three quality control samples in the same way. The calculated RSD values from repeated measurements were summarized in Table 2.

### 3.4. Accuracy

The accuracy of the method was demonstrated at three different concentration levels (50–150%) by spiking a known quantity of DEX sodium phosphate, OND hydrochloride, GRA hydrochloride, TRO hydrochloride, and AZA hydrochloride standard drugs into infusion samples in triplicate. The results for accuracy of the method are given in Table 3. Recoveries of DEX sodium phosphate, OND hydrochloride, GRA hydrochloride, TRO hydrochloride, and AZA hydrochloride in infusion samples were between 98.0 and 102.0%, indicating the good accuracy of the developed method.

### 3.5. Physicochemical Stability Study of the Infusion Samples

**Figure 3:**
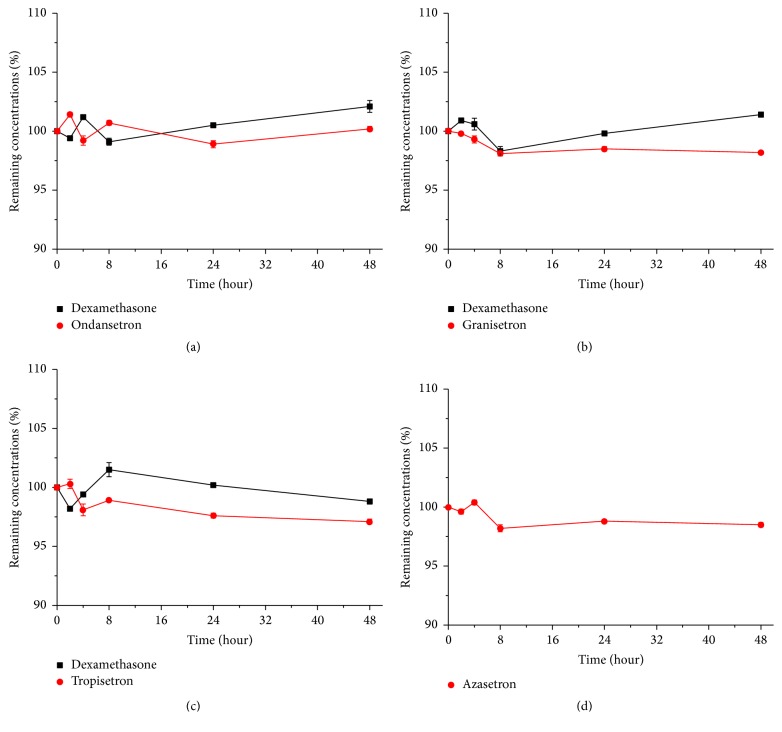
Drug concentrations (mean ± SD [%]; *n* = 3) of DEX-OND, DEX-GRA, DEX-TRO, and AZA in 0.9% sodium chloride injection over 48 hours at 25°C.

No precipitation or color change was observed in all of the infusion mixtures during the 48 hours storage period. The results of the HPLC analysis for each of the test drugs are presented in Figure 3. There was no loss of DEX sodium phosphate that occurred with any of the drugs over 48 hours stored at 25°C. Similarly, little or no loss of OND hydrochloride, GRA hydrochloride, TRO hydrochloride, and AZA hydrochloride occurred over 48 hours. The average pH of the infusion mixtures stored at 25°C over 48 hours measurements are given in Table 4. The pH of all infusions was considered insignificant over 48 hours. Based on the data presented herein, we believe that the mixtures of DEX sodium phosphate-OND hydrochloride; DEX sodium phosphate-GRA hydrochloride; DEX sodium phosphate-TRO hydrochloride and AZA hydrochloride in normal saline were stable for up to 48 hours when stored in polyolefin bags protected from daylight at 25°C. The satisfactory stability of the drugs in the mixtures makes it possible to prepare them in advance by licensed central intravenous additive services, which may be convenient in hospitals.

## 4. Conclusions

The developed HPLC-DAD method is simple, sensitive, specific, and adequate to the simultaneous quantification of DEX sodium phosphate, OND hydrochloride, GRA hydrochloride, TRO hydrochloride, and AZA hydrochloride in infusion samples. The method was successfully applied to a study of the chemical stability of these infusion mixtures under 25°C for 48 hours. The method might also be suitable for application to other analytical problems, for example, quality control of pharmaceutical formulations or evaluating the chemical stability of referred drugs in mixtures for clinical use. The results of the stability study showed that mixtures of DEX sodium phosphate-OND hydrochloride; DEX sodium phosphate-GRA hydrochloride; DEX sodium phosphate-TRO hydrochloride and AZA hydrochloride in normal saline in 0.9% sodium chloride injection when stored in polyolefin bags protected from daylight were chemically stable for 48 hours at 25°C.

## Figures and Tables

**Table 1:** System suitability parameters for DEX, OND, GRA, TRO, and AZA.

Analytical parameter	DEX	OND	GRA	TRO	AZA
Detection wavelength (nm)	241	310	302	285	302
Retention time (min)	10.72	8.12	5.97	7.10	4.55
Theoretical plate (mean ± SD)	8567 ± 129	9476 ± 98	9075 ± 104	9593 ± 115	8445 ± 112
Linear range (mg/L)	1.0–100.0	2.0–80.0	1.2–36.0	1.0–50.0	2.0–100.0
Linear equation	*Y* = 0.7519*X* − 0.1208	*Y* = 1.5213*X* − 6.444	*Y* = 0.7563*X* + 0.3852	*Y* = 0.6965*X* + 0.7363	*Y* = 0.1918*X* + 0.1513
Correlation coefficient (*r*)	0.9992	0.9999	0.9997	0.9995	0.9998
Quantification limit (mg/L)	0.1	0.3	0.18	0.15	0.6
Detection limit (mg/L)	0.04	0.1	0.06	0.05	0.2

**Table 2:** Precision of the method.

Compound	Concentration tested (mg/L)	Precision RSD (%)	
		Intraday	Interday
DEX sodium phosphate	1	1.31	1.74
	20	0.72	0.95
	40	0.45	0.71
OND hydrochloride	2	0.62	1.53
	16	0.70	1.54
	32	1.54	2.16
GRA hydrochloride	1.2	1.06	2.28
	6	1.03	1.35
	12	0.41	1.90
TRO hydrochloride	1	0.55	1.28
	10	1.25	1.96
	20	0.42	1.15
AZA hydrochloride	2	1.80	2.47
	20	0.86	1.52
	40	0.34	1.15

**Table 3:** Accuracy of the method.

Compound	Amount added (mg/L)	Amount recovered (mean ± SD, mg/L)	% recovery (mean ± SD, *n* = 3)
DEX sodium phosphate	10	9.988 ± 0.166	99.88 ± 1.66
	20	19.897 ± 0.526	99.48 ± 2.63
	30	30.242 ± 0.579	100.81 ± 1.93
OND hydrochloride	8	7.878 ± 0.142	99.48 ± 1.78
	16	16.174 ± 0.138	100.09 ± 0.86
	24	23.895 ± 0.403	99.56 ± 1.68
GRA hydrochloride	3	3.764 ± 0.021	99.69 ± 0.70
	6	6.013 ± 0.062	100.21 ± 1.03
	9	9.078 ± 0.111	100.87 ± 1.24
TRO hydrochloride	5	4.956 ± 0.068	99.13 ± 1.36
	10	9.876 ± 0.166	98.76 ± 1.66
	15	14.860 ± 0.122	99.06 ± 0.81
AZA hydrochloride	10	10.018 ± 0.129	100.18 ± 1.29
	20	19.979 ± 0.486	99.89 ± 2.43
	30	30.326 ± 0.246	101.09 ± 0.82

**Table 4:** pH values (mean ± SD; *n* = 3) of the infusion samples over 48 hours.

Time (hours)	pH value			
	DEX + OND	DEX + GRA	DEX + TRO	AZA
0	5.65 ± 0.01	5.52 ± 0.04	6.42 ± 0.02	4.91 ± 0.05
2	5.72 ± 0.04	5.43 ± 0.02	6.49 ± 0.04	4.85 ± 0.02
4	5.77 ± 0.03	5.55 ± 0.01	6.51 ± 0.04	4.83 ± 0.01
8	5.73 ± 0.01	5.57 ± 0.01	6.46 ± 0.01	4.86 ± 0.06
24	5.62 ± 0.02	5.50 ± 0.05	6.55 ± 0.02	4.80 ± 0.03
48	5.70 ± 0.01	5.58 ± 0.02	6.54 ± 0.02	4.83 ± 0.02
